# Risk factors and predictive markers of post-stroke cognitive decline–A mini review

**DOI:** 10.3389/fnagi.2024.1359792

**Published:** 2024-02-13

**Authors:** Xiaofan Guo, Cattien Phan, Sanad Batarseh, Miao Wei, Justin Dye

**Affiliations:** ^1^Department of Neurology, Loma Linda University, Loma Linda, CA, United States; ^2^Department of Neurosurgery, Loma Linda University, Loma Linda, CA, United States

**Keywords:** stroke, cognitive decline, acute stroke intervention, cerebrovascular disease, ageing

## Abstract

Stroke is one of the top causes of death and disability worldwide. Cognitive impairments are found in more than 70% of individuals who have survived a stroke. Cognitive decline is a major contributor to disability, dependency, and morbidity. The prevalence and severity of dementia vary depending on different characteristics of the stroke and other clinical risk factors. Here we discuss the effects of stroke territory, patients’ age, sex, cerebral blood flow, acute reperfusion therapy, and cognitive reserve of post-stroke cognitive decline. Potential predictive molecular and genetic biomarkers of post-stroke cognitive impairments are also discussed.

## Introduction

Stroke, a prevalent cerebrovascular disorder, significantly contributes to mortality and long-term disability (Rost et al., 2022). The advancement of effective acute interventions and stroke rehabilitation has led to global improvements in stroke outcomes (Goyal et al., 2016; Le Danseur, 2020). However, post-stroke cognitive decline (PSCD) is still prevalent and contributes significantly to disability. Previously, researchers classified post-stroke cognitive decline as vascular dementia (Sun et al., 2014). However, not all stroke survivors experiencing cognitive decline meet the criteria for dementia. Consequently, more attention was put on PSCD instead. PSCD has been reported to result from various stroke types, such as ischemic stroke, intracerebral hemorrhage, and aneurysmal subarachnoid hemorrhage (Rost et al., 2022). Studies have indicated that stroke escalates the risk of cognitive impairment by a significant factor of five to eight times (Kulesh et al., 2018) and cognitive deficits are found in over 70% of stroke patients. PSCD negatively impacts motor function recovery and recovery of independent activities of daily living in these patients. It increases the challenges of comprehensive rehabilitation, hindering patients from reintegrating into family and society. A growing body of research indicates that cognitive function can serve as a predictor for the functional outcomes of stroke patients (Almalki et al., 2018). Considering the prevalence and diverse array of factors involved in triggering or exacerbating cognitive dysfunction in stroke patients, identifying the risk factors and biomarkers that contribute to cognitive decline after stroke has the potential to guide the development of targeted therapies and inform strategies for preventing cognitive impairment in the future. Here we discuss the effect of stroke territory, patients’ age, sex, cognitive reserve, acute reperfusion therapy, and cerebral blood flow on cognitive decline. As predictive markers, potential molecular and genetic biomarkers of post-stroke cognitive impairments are also discussed.

## Clinical features of post-stroke cognitive decline

Post-stroke cognitive dysfunction (PSCD) is characterized as any cognitive impairments that manifest after a stroke, regardless of the underlying cause of the stroke (Quinn et al., 2021). The dysfunction is not only confined to the direct impairment of the function of stroke territories like aphasia, apraxia, acalculia, and memory deficits (Rundek et al., 2022) but also other higher-order cognitive functions. These functions include orientation, visuospatial abilities, executive function, attention, and other aspects, unrelated to the stroke territories. There is no consensus on the terms used to describe cognitive disorders after stroke. The International Society for Vascular Behavioral and Cognitive Disorders and the VICCS (Vascular Impairment of Cognition Classification Consensus Study) guidelines utilize mild vascular cognitive disorder, vascular dementia, or major vascular cognitive disorder (Skrobot et al., 2018). Meanwhile, the Diagnostic and Statistical Manual of Mental Disorders-5 (DSM-5) employed the terms mild and major neurocognitive disorder. On the other hand, the International Classification of Diseases, Tenth Revision (ICD-10), lacks a specific definition for either PSCD but does include a definition for vascular dementia. Cognitive screening tools have been used to evaluate stroke patients and diagnose PSCD. However, those tests require intact verbal expression and comprehension as well as hearing and vision, which are often impaired in left-hemisphere stroke patients. The Mini Mental State Examination (MMSE) or the Montreal Cognitive Assessment test (MoCA) are the most utilized cognitive screening tools. However, they are not sensitive to higher-level attentional issues like mood and behavioral measures, which significantly influence activities of daily living (Rost et al., 2022). Developing a better cognitive screening tool is necessary for better identifying cognitive decline in stroke patients.

## Age of patients

A well-known major risk factor for the development of dementia is increasing age given that elderly people are prone to experience memory loss and cognitive slowing in multiple domains that can interfere with everyday activities (Lo Coco et al., 2016). At the same time, stroke and dementia have an intricate relationship with many shared characteristics, one of which is age. There are several studies that showed age is a risk factor for stroke and PSCD. In addition to the Reasons for Geographic and Racial Differences in Stroke (REGARDS) study (Howard et al., 2005), a prospective cohort study conducted over 10 years showed improved global cognition over a 10-year period after the onset of the first stroke in young stroke survivors in comparison to the older group, which showed an ongoing gradual decline (Elgh and Hu, 2019). Of the 38 patients that participated in the study, 79% had an ischemic stroke, 16% had a hemorrhagic stroke, and the remaining 5% were unknown. When comparing between ischemic and hemorrhagic strokes, no significant differences were observed. An individual participant data meta-analysis published in 2021 also identified similar results. The study identified age as a significant risk factor for PSCD in global cognition and executive function and showed that an increased rate of decline after stroke is associated with older age (Lo et al., 2022). The SONIC study further quantified this relationship and showed an approximate one-fold increase in risk of PSCD with an age increase of 1 year (Srithumsuk et al., 2020). These studies all support the idea that neuropsychological and cognitive assessment should be a part of stroke prevention and post-stroke care in both the short and long term with great efforts directed toward a multidisciplinary approach. This approach should include assessment and discussion of quality of life along with caregiver support (Renjen et al., 2015). As a result, clinicians should pay more attention to monitoring cognitive function in older stroke patients and give treatment in the early stages to decelerate the advancement of dementia.

## Gender of the patients

Another significant risk factor for stroke and PSCD is sex differences across multiple areas, particularly post-stroke outcomes, such as quality of life and mortality. In general, women have a higher prevalence of lifetime risk of cerebrovascular diseases, dementia, and post-stroke functional outcomes (Wyller et al., 1997; Seshadri and Wolf, 2007; Sohn et al., 2018; Levine et al., 2021). It has been shown that the severity of stroke is worse in women with a 1-month case fatality of 24.7% in comparison to men with 19.7% (Appelros et al., 2009). Outcomes after ischemic stroke are worse for women in both short-term and long-term intervals. A study in 2020 looking at sex differences in post-stroke cognitive outcomes found that women had worse cognitive outcomes 3 months after a stroke and identified major contributing factors to be older age at stroke onset, higher prevalence of widowhood, worse pre-stroke functional and cognitive status, and lower educational background (Dong et al., 2020). Another study assessing the quality of life after an ischemic stroke showed that women tend to have worse quality of life compared to men up to 12 months after stroke (Bushnell et al., 2014). The INTERACT1 study looked at PSCD in intracerebral hemorrhage (ICH) for 90-day survivors and found that female sex was one of the factors that were independently associated with cognitive impairment (You et al., 2017). In contrast, a recent study published in 2023 found that the prevalence of post-stroke cognitive impairment is similar between men and women. However, the cognitive domains affected differ with men at higher risk for verbal memory impairment and women at higher risk of impairment in language, attention, and executive function (Exalto et al., 2023). Interestingly, the study also found that the MMSE has higher sensitivity but lower specificity for women. Therefore, it is important to understand the sex differences in PSCD to refine standard guidelines of post-stroke care and better address the unique needs of certain populations at higher risk.

## Stroke territory

Stroke affecting different territories has significantly varying clinical symptoms, including hemineglect, language, visual, motor, and sensory deficits. With post-stroke cognitive impairment occurring in approximately half of people in the first year after a stroke, further investigation into the correlation of cognitive decline and the vascular territory involved can help categorize patients at the highest risk. This could then provide more comprehensive screening and earlier diagnosis. A recent study attempted to assess the correlation of patients with a lower Alberta Stroke Program Early CT Score (ASPECTS) to predict post-stroke cognitive impairment, showing a direct correlation of cognitive impairment with worsened ASPECT scores (Esmael et al., 2021). Another study assessing patients in the acute phase of stroke found that domains involving naming, language, and delayed recall were significantly decreased only in patients with left sided cerebral infarctions (Chu et al., 2023). A large-scale multicohort lesion-symptom mapping study, sampling 2,950 patients where PCSD was defined as performance lower than the fifth percentile of local normative data on at least one cognitive domain on a Montreal Cognitive Assessment, found strong associations of PSCD with infarcts in the left frontotemporal lobes, left thalamus, and right parietal lobe (Weaver et al., 2021). The ICONS study further supported this correlation finding thalamic lesions significantly correlated with cognitive impairment, even in younger patient populations (Zhang et al., 2022). With posterior circulation structures, a study found significant cognitive impairment in patients with cerebellar infarcts; specifically a higher incidence of significant impairment with right cerebellar lesions showing significantly impaired executive function (Liu et al., 2022). Similar correlations in intracerebral hemorrhage location and resultant cognitive impairment post-stroke were observed in a prospective study that found a strong correlation where patients with lobar hematoma were 4.58 times more likely than patients with non-lobar ICH (Li et al., 2023). Further correlation localizing areas where cognitive impairment is significant would potentially be beneficial for making post-stroke care more individualized.

## Baseline cerebral blood flow condition

Intracranial atherosclerotic stenosis (ICAS) is defined as > 50% stenosis and is an important risk factor for both ischemic stroke and cognitive impairment. Unstable plaques can lead to symptomatic ICAS, resulting in ischemic infarcts, while asymptomatic ICAS is linked to cognitive decline with an unknown pathogenesis (Si et al., 2022). A recent study even showed that carotid revascularization has been demonstrated to lead to cognitive improvements, most significantly in younger patients with the most pronounced impairments (Turowicz et al., 2021). There has also been a correlation between chronic heart failure and cognitive decline, with the proposed mechanism being chronic hypoperfusion to critical brain areas. Given the causation of stroke and the correlation of cognitive decline, PSCD in patients with ICAS is important to assess, as more ischemic stroke patients are falling into this category. A cross-sectional study of minor stroke patients using the MoCA demonstrated that patients with ICAS were more likely to develop cognitive impairment after an acute, non-disabling ischemic stroke compared to their counterparts without ICAS (Schaap et al., 2002). A *post hoc* analysis of the Stenting and Aggressive Medical Management for Preventing Recurrent Stroke in Intracranial Stenosis (SAMMPRIS) trial assessed 393 patients who met the inclusion criteria, showing a suggestion of anterior circulation ICAS correlating with worsening MoCA scores as well (Yaghi et al., 2020). As a result, when surgeons are evaluating the indications for carotid intervention in patients who also have ICAS, cognitive decline is one more factor they may want to consider.

## Acute reperfusion therapy

The research on the impact of acute reperfusion therapy including endovascular thrombectomy (EVT) and intravenous thrombolysis (IVT) is limited. In 2017, a study performed in Sweden that enrolled 75 patients with first-time ischemic stroke received either EVT or IVT at the Sahlgrenska University Hospital in Gothenburg from 2009–2010. At the 6-year mark following a stroke, participants underwent cognitive screening using the Montreal Cognitive Assessment (MoCA). A total of 33.3% of patients in the IVT group and 50% of patients in the EVT group had a MoCA score lower than 26 (A MoCA score < 26 indicates cognitive impairment), which indicates cognitive decline persists even after reperfusion (Muhr et al., 2017). However, a recent study performed in Italy by Lattanzi et al. (2020) showed promising data. A total of 88 patients (average age of 66.3 ± 12.9 years) were enrolled and 38 of them received treatment with IVT alone, while 50 underwent a combination of IVT and EVT. Six months after experiencing an anterior circulation ischemic stroke, individuals who received both EVT and intravenous IVT demonstrated better cognitive performance than those who only underwent IVT (Lattanzi et al., 2020). However, both of these studies were performed in a single center with a relatively small sample size. Future studies with bigger sample sizes and multi-center designs are needed to evaluate the impact of reperfusion therapy on cognitive decline. Another related topic is whether it is worth performing EVT on patients with premorbid cognitive decline. After analyzing multiple observational studies, encompassing both case series and registry-based investigations, the American Heart Association/American Stroke Association published a scientific statement in 2022 that EVT therapy in patients with premorbid dementia has comparable safety compared to those without (Ganesh et al., 2022).

## Cognitive reserve

The theory of Cognitive Reserve (CR) describes individual differences, such as enriched environments and life experiences, in susceptibility to both age-related brain changes and pathologic changes (Stern, 2012). The cognitive reserve hypothesis suggests that people with high cognitive reserve can process and perform tasks in a way that makes them more resilient to neuropathological changes. This concept has been applied to understanding dementia, such as Alzheimer’s dementia. Only recently has the effect of cognitive reserve been discussed with stroke and PSCD. The Korean Stroke Cohort for Functioning and Rehabilitation (KOSCO) longitudinal study showed that lower cognitive reserve specifically focused on lower premorbid education and occupation, was associated with severe cognitive dysfunction and an increased risk of cognitive impairment immediately and over the following 30 months after the stroke (Shin et al., 2020). It also found that a higher level of education or occupation was associated with a faster recovery. Additionally, a recent study done in 2022 showed that patients with a higher level of CR had better cognitive function after experiencing an acute ischemic stroke (Li et al., 2022). These findings suggest a protective effect with higher CR. Data on CR and PSCD for hemorrhagic stroke or ICH is limited. While additional studies are likely needed to better understand the relationship between CR and PSCD, the existing evidence suggests that the effect of CR on cognitive function after stroke is significant and may play a large role in stroke recovery. It also highlights the potential of preventing PSCD by recognizing inter-individual variability and offering a more structured cognitive management strategy to those with increased risks.

## Molecular markers and genetic contributions

Currently, standard practice is to use neuropsychological testing to assess and diagnose PSCD, which has its limitations due to its subjectivity and limited prognostication ability. An increased number of studies have investigated the expression of biomarkers such as C-reactive protein (CRP), interleukin 6 (IL-6), and interleukin 10 (IL-10). In 2023, a Meta-Analysis that involved nine studies with 3,893 participants showed the concentrations of peripheral CRP, especially in the acute stroke phase, are significantly increased for patients with cognitive decline after stroke compared to the patients without cognitive decline (Wang et al., 2023). A prospective study conducted in 2022 that involved 1,003 stroke patients indicated elevated IL-6 levels were independently linked to a decline in MoCA scores following ischemic stroke and transient ischemic attack (TIA) (Wang et al., 2022). Another study suggested that homocysteine (Hcy), total cholesterol (TC), and low-density lipoprotein cholesterol (LDL-C) also can serve as possible biomarkers in patients with post-stroke cognitive impairment (Kim et al., 2022). Additionally, neurofilament light (NfL), a neuronal cytoplasmic protein, has also been found to be associated with long-term cognitive function after stroke. A longitudinal study that recruited 304 patients with PSCD divided patients into a progression group (as determined by decreased Telephone Interview of Cognitive Status-40 (TICS-40) scores) and a stable group (as determined by increased or unchanged TICS-40 scores). Between the two divided groups, the group with progressively worsening TICS-40 scores had significantly higher serum NfL levels (Wang et al., 2021). A variety of biomarkers, including those with a pathogenic correlate, have continued to be studied, with samples from CSF, serum, and urine. Studies on the genetic profile of PSCD patients have also been performed. Sun et al. (2015) performed a meta-analysis study that studied genetic polymorphism in 4,462 vascular dementia patients and 11,583 controls and identified vascular dementia is associated with APOE ϵ2/ϵ3/ϵ4 and four additional genetic polymorphisms, namely MTHFR C677T, PON1 L55M, TGF-β1 + 29C/T, and TNF-α -850C/T. In addition, a recent population-based cohort study, among 1,767 genotyped patients with stroke or TIA showed APOE-ε4 homozygosity was associated with both pre- and post-vascular events of dementia (Pendlebury et al., 2020). Individuals of this genotype exhibited a 2.9-fold rise in the risk of dementia within a 5-year period. Interestingly, the evidence suggests that PSCD and late-onset Alzheimer’s disease may share the same genetic risk factor, APOE-ε4 (Kunkle et al., 2019). Finding reliable biomarkers in acute stroke patients may improve early detection of PSCD.

## Discussion

With the aging of the global population, the 2022 Global Stroke Factsheet indicates that the likelihood of experiencing a stroke in one’s lifetime has risen by 50% in the past 17 years. As one of the most disabling complications after stroke, PSCD has drawn more attention from physicians and scientists. Identifying the risk factors, discussed in this review may help clinicians predict PSCD after stroke. Vascular risk factors like high blood pressure, smoking, diabetes, high alcohol consumption, elevated LDL, and APOE-ε4 mutation are associated with both increased risk of stroke and dementia. This implies the strong connection between stroke and cognitive decline. As a result, the best way to prevent PSCD is primary stroke prevention by modifying stroke risk factors, which will both reduce the risk of stroke and dementia. However, there is no effective pharmacological treatment for PSCD or vascular dementia so far. In 2021, there was a meta-analysis that enrolled 4,373 participants to measure the efficiency of cholinesterase inhibitors for vascular dementia and other PSCD. The results demonstrated that there is a slight beneficial effect of donepezil and galantamine on cognition, but this effect is unlikely to be clinically important (Battle et al., 2021). Other trials also failed to exhibit significant benefits of cholinesterase inhibitors or memantine in improving cognitive decline caused by stroke (Kavirajan and Schneider, 2007; Narasimhalu et al., 2010; McShane et al., 2019). However, in stroke patients who have progressive cognitive decline that is not directly attributable to a clinical stroke, cholinesterase inhibitor therapy is still indicated. The idea of cognitive rehabilitation has been raised recently (Lanctot et al., 2020), but no clinical trials have proven its benefits yet. Although we have identified multiple risk factors and potential modifiers, more efforts should be put into the management and prevention of PSCD in future studies to improve the comprehensive recovery of stroke patients.

Due to the less frequent occurrence of ICH compared to ischemic stroke and its elevated mortality rate, much of our understanding of post-stroke cognitive impairment has traditionally concentrated on the ischemic stroke subtype. However, a nationwide population-based cohort study performed in Danish, which compared 84,220 ischemic stroke patients, 16,723 intracerebral hemorrhage patients, and 9,872 subarachnoid hemorrhage patients, indicates the risk of post-stroke cognitive decline for ICH patients was higher than ischemic stroke patients (Corraini et al., 2017). A retrospective study that enrolled 184 ICH patients showed as high as 84% of patients developed cognitive decline after hemorrhage (Banerjee et al., 2018). Another recent prospective study published in 2023 identified early cognitive impairment as prevalent among ICH patients and significantly decelerated the recovery of functional outcomes in patients (Li et al., 2023). As a result, in ICH patients, conducting early cognitive assessments will also be crucial in formulating personalized predictions, targeted interventions, and rehabilitation strategies. Although there is a high prevalence of ICH-associated cognitive decline, their management and prevention of are still not clear and more studies in this field are needed in the future.

## Author contributions

XG: Data curation, Investigation, Methodology, Writing—original draft. CP: Writing—original draft. SB: Writing—original draft. MW: Writing—original draft. JD: Conceptualization, Supervision, Writing—review and editing.
